# Upregulated antimicrobial immune response mediated by neutrophils in the development from allergic rhinitis to asthma

**DOI:** 10.3389/fimmu.2022.1026121

**Published:** 2022-12-08

**Authors:** Lisha Li, Hao Zhang, Xiujie Wang, Zixi Wang, Le Cui, Yingyang Xu, Kai Guan

**Affiliations:** ^1^ Department of Allergy, Beijing Key Laboratory of Precision Medicine for Diagnosis and Treatment on Allergic Diseases, National Clinical Research Center for Dermatologic and Immunologic Diseases, Peking Union Medical College Hospital, Chinese Academy of Medical Sciences & Peking Union Medical College, Beijing, China; ^2^ Institute of Genetics and Developmental Biology, Innovation Academy of Seed Design, Chinese Academy of Sciences, Beijing, China; ^3^ School of Future Technology, University of Chinese Academy of Sciences, Beijing, China

**Keywords:** asthma, allergic rhinitis, transcriptome, neutrophils, natural killer cells, antimicrobial peptides

## Abstract

**Background:**

Allergic rhinitis (AR) and asthma are closely related, and AR is regarded as an important risk factor for the onset of asthma. However, the pathogenesis of the development of asthma from AR is still undefined.

**Objective:**

The aim of this study was to investigate the mechanisms underlying the development of asthma from AR by comparing the transcriptome features of patients with AR with and without asthma.

**Methods:**

Patients with AR with or without asthma caused by weed pollen who presented to the Allergy Clinic of Peking Union Medical College Hospital were recruited for this study. Peripheral blood samples of all the patients were collected during the weed pollen season (September) when the patients had allergic symptoms and outside the pollen season (November) when the patients had no symptoms. Transcriptomic analysis was conducted, and the differentially expressed genes (DEGs) and enriched immune pathways between the patients with AR with asthma (AR-asthma group) and those without asthma (AR group) were identified. In addition, the expression levels of some pivotal differentially expressed RNAs were quantified using quantitative polymerase chain reaction (PCR).

**Results:**

During the weed pollen season, the immune-related Gene Ontology (GO) terms with *P* value < 0.05, enriched by the upregulated genes in the AR-asthma group compared to the AR group included antifungal humoral response, neutrophil-mediated killing of bacterium, antibacterial humoral response, antimicrobial humoral immune response mediated by antimicrobial peptides, and regulation of the T cell receptor signaling pathway. The immune-related GO terms with *P* values <0.05 enriched by downregulated genes were positive regulation of natural killer cell-mediated cytotoxicity, microglial cell activation, natural killer cell activation, and leukocyte-mediated cytotoxicity. The GO term of antimicrobial humoral immune response mediated by antimicrobial peptides was upregulated both during and outside the pollen season, and the upregulated expression of three DEGs (LTF, PF4, and ELANE) included in this term was verified through quantitative PCR.

**Conclusions:**

The activation of the antimicrobial immune response mediated by neutrophils and the depression of cytotoxicity mediated by natural killer cells may play roles in the progression from AR to asthma.

## Introduction

Allergic rhinitis (AR) is a common allergic airway disease that affects 14% of adults in the United States (1) and approximately 17.6% of people in major Chinese cities (2). The incidence of epidemiologic AR in the grasslands of northern China is 32.4%, and the most common allergen is weed pollen (3). Asthma is also a common airway disease and its prevalence ranges between 5% and 16% worldwide (4). It is well known that AR and asthma frequently coexist and are closely related because of their shared physiology and pathology (5). In addition, it has been reported that AR is an important risk factor for the onset of asthma. In a 10-year retrospective study of 436 participants, diagnosis of AR at baseline was found to be a significant predictive factor for the development of asthma at the end of follow-up with the OR of 7.8 (6). Yin et al. investigated the natural course of AR caused by weed pollen in 1096 patients and found that 37% of the patients developed asthma within 5 years (7). Both AR and asthma severely affect the quality of life of patients and exert heavy financial burdens on patients, their families, and the society (8, 9).

The pathogenesis underlying the development of asthma from AR is unclear. Some authors have discussed the predictors for the progression from AR to asthma and reported that cigarette smoking, female sex, and bronchial hyper-responsiveness are possible risk factors for the onset of asthma in patients with AR (10–12). Panganiban et al. (13) identified 30 circulating microRNAs that are differentially expressed among healthy controls, patients with AR, and patients with asthma. In that study, miR-125b, miR-126, miR-21, miR-16, miR-223, miR-148a, and miR-146a were upregulated in the asthma group compared to the AR or healthy groups. Sobkowiak et al. (14) found that the expression of four proteins associated with airway fibrosis is significantly different between children with AR and those with asthma. In another study of patients with dust mite allergy, patients with asthma had higher neutrophil counts and IL-8 levels in the sputum than those without asthma both at baseline and 24 h after bronchial allergen challenge; however, the sputum eosinophil count and eosinophil cationic protein levels of the two groups were indistinguishable (15). However, more intensive studies are still necessary for the acquisition of repeatable and consistent results and the clarification of the specific mechanism of the progression from AR to asthma.

The aim of this study was to investigate the mechanisms underlying the development of asthma from AR by comparing the features of gene expression through transcriptome sequencing between patients with weed pollen induced AR and asthma and patients with weed pollen induced AR only. Because of the existence of persistent inflammation in the airway of patients with seasonal AR and asthma during and outside the pollen season (16, 17), the transcriptomic data both during and outside the season will be analyzed.

## Materials and methods

### Subjects

All the patients included in this study were recruited from the Allergy Clinic of Peking Union Medical College Hospital. The inclusion criteria were as follows: 1) adult patients; 2) patients with typical symptoms of rhinitis (18), including rhinorrhea, sneezing, and itchy nose and eyes, with or without asthma, during the weed pollen season (from August to September in Northern China); 3) positive results of intradermal tests for one or more kinds of weed pollen, including artemisia, humulus, and chenopodium pollen (diameter of wheal ≥10 mm); or specific immunoglobulin E (IgE) level to one or more kinds of weed pollen ≥ 0.7 KUA/L (ImmunoCAP system, Thermo Fisher Scientific, US). Concomitant asthma in patients with AR was diagnosed based on typical respiratory symptoms and variable expiratory airflow limitations during the weed pollen season, according to the criteria of the Global Initiative for Asthma report (19). The exclusion criteria were as follows: 1) pregnant or lactating women and 2) patients with chronic infections, immunodeficiency diseases, autoimmune disorders, or tumors. This study was approved by the Peking Union Medical College Hospital Review Board (ZS-1917). All patients provided written informed consent for participation in this study.

### Study design

This was a cross-sectional study conducted during the autumn and winter seasons of 2020. Peripheral blood samples of the patients were collected at two time points: during the weed pollen season, when all the patients had AR symptoms with or without concomitant asthma (September), and after the weed pollen season, when all the patients had no allergic symptoms (November). Transcriptomic analysis of all blood samples was conducted, and the transcriptomic features of the patients with AR and asthma (AR-asthma group) were compared with those of patients with AR only (AR group) during and outside the pollen season. The expression levels of some pivotal differentially expressed ribonucleic acids (RNAs) identified through transcriptome sequencing were further quantified using quantitative polymerase chain reaction (qPCR).

### Transcriptomic RNA sequencing and bioinformatics analysis

Total RNA was isolated from the blood sample which was stored at −80 °C. Messenger RNA (mRNA) was purified from total RNA using polyT and fragmented into 300–350 bp fragments. First-strand complementary deoxyribonucleic acids (cDNAs) were reverse-transcribed with fragmented RNA and deoxynucleotide triphosphates (dNTPs), and second-strand cDNA synthesis was subsequently performed. After adenylation of the 3’ ends of the DNA fragments, sequencing adaptors were ligated to the cDNA and the library fragments were purified. The template was enriched using PCR, and the PCR product was purified to obtain the final library. After library construction, high-throughput sequencing was performed using the Illumina Novaseq6000 sequencing platform (Illumina, San Diego, CA, USA).

For quality control of sequencing data, we analyzed the quality of raw data using FastQC v0.11.5 and obtained clean reads after removal of low-quality (quality score < 20) and adaptor sequences using Cutadapt v2.7. Thereafter, clean reads were mapped to the human reference genome GRCh38 using HISAT2 v2.1 (20). Uniquely mapped reads were utilized for the quantification of gene expression, which was performed using featureCounts v2.0.0 (21). Expressed genes were obtained by setting average fragments per kilobase of transcript per million fragments mapped (FPKM) ≥ 1 across all samples, followed by identification of differentially expressed genes (DEGs) within these genes using DESeq2 v1.30.1 (22) under the condition of |fold change| ≥ 1.2 and *P* value < 0.05. Gene Ontology (GO) enrichment analysis of immune-related process was conducted using ClueGO v2.5.8 (23) in Cytoscape v3.9.0 with “GO-ImmuneSystemProcess-EBI-UniProt-GOA-ACAP-ARAP-15.02.2022” as the input ontology file and visualized with ggplot2 v3.3.2. Then the GO terms were ordered based on the rich factor, which is calculated by number of genes enriched in a specific item divided by total number of genes in this term.

### Quantitative PCR

Reverse transcription was conducted with the PrimeScript RT reagent Kit with gDNA Eraser for RT-PCR (RR047A, Takara Bio Inc., Beijing, China) and the thermal cycler (Thermo Fisher Scientific, Waltham, MA, USA) in a volume of 20 µL. The single cycle was 35 °C for 15 min and the incubation period was 85 °C for 5s. Relative qPCR was done with the Bio-Rad CFX Opus 96 Instrument and TB Green® Premix (RR820A, Takara Bio Inc., Beijing, China). Fold induction was calculated using the comparative Ct method and the formula 2^-(ΔΔCt).^

### Statistical analysis

Normally distributed data are expressed as mean and standard deviation, while non-normally distributed data are expressed as median and interquartile range. Pearson χ2 test was used to compare sex ratios at baseline, whereas the differences in patient body mass index (BMI), length of AR course, and visual analog scale (VAS) scores between the AR and AR-asthma groups were verified using the Mann-Whitney U test. The expression levels of critical DEGs measured using quantitative PCR were compared between the two groups using t-tests. Statistical significance was set at *P <* 0.05. All statistical analyses were performed using SPSS statistical software (v23; SPSS Inc., Chicago, IL, USA).

## Results

### Demographic characteristics and clinical manifestations of the patients

Twenty-five patients with AR allergic to weed pollen were recruited for this study. Of these, seven patients had concomitant asthma. As shown in **Table 1**, there were no significant differences in age distribution, male-to-female ratio, BMI, length of AR course, and VAS score of AR symptoms in the previous pollen season between the AR (n = 18) and AR-asthma (n = 7) groups. However, the VAS score of the AR-asthma group tended to be higher than that of the AR group (7.5 [7.0, 9.0] vs. 7.0 [5.0, 8.0]; *P* = 0.087).

**Table 1:** Comparison of baseline characteristics between the AR group and AR-asthma group.

Indexes	AR group (n=18)	AR-asthma (n=7)	*P* value
Women (n, %)	9, 50.0%	3, 42.9%	1.000
Age (years old)	34 ± 8	32 ± 9	0.551
BMI (kg/m^2^)	22.8 (20.6, 24.2)	26.4 (22.1, 30.0)	0.064
Course of AR (years)	5 (3, 9)	7 (4, 9)	0.745
VAS of past AR symptoms	7.0 (5.0, 8.0)	7.5 (7.0, 9.0)	0.087

AR, allergic rhinitis; BMI, body mass index; VAS, visual analogue scale.

### Transcriptomic differences between the AR-asthma and AR groups during the pollen season

**Figure 1:**
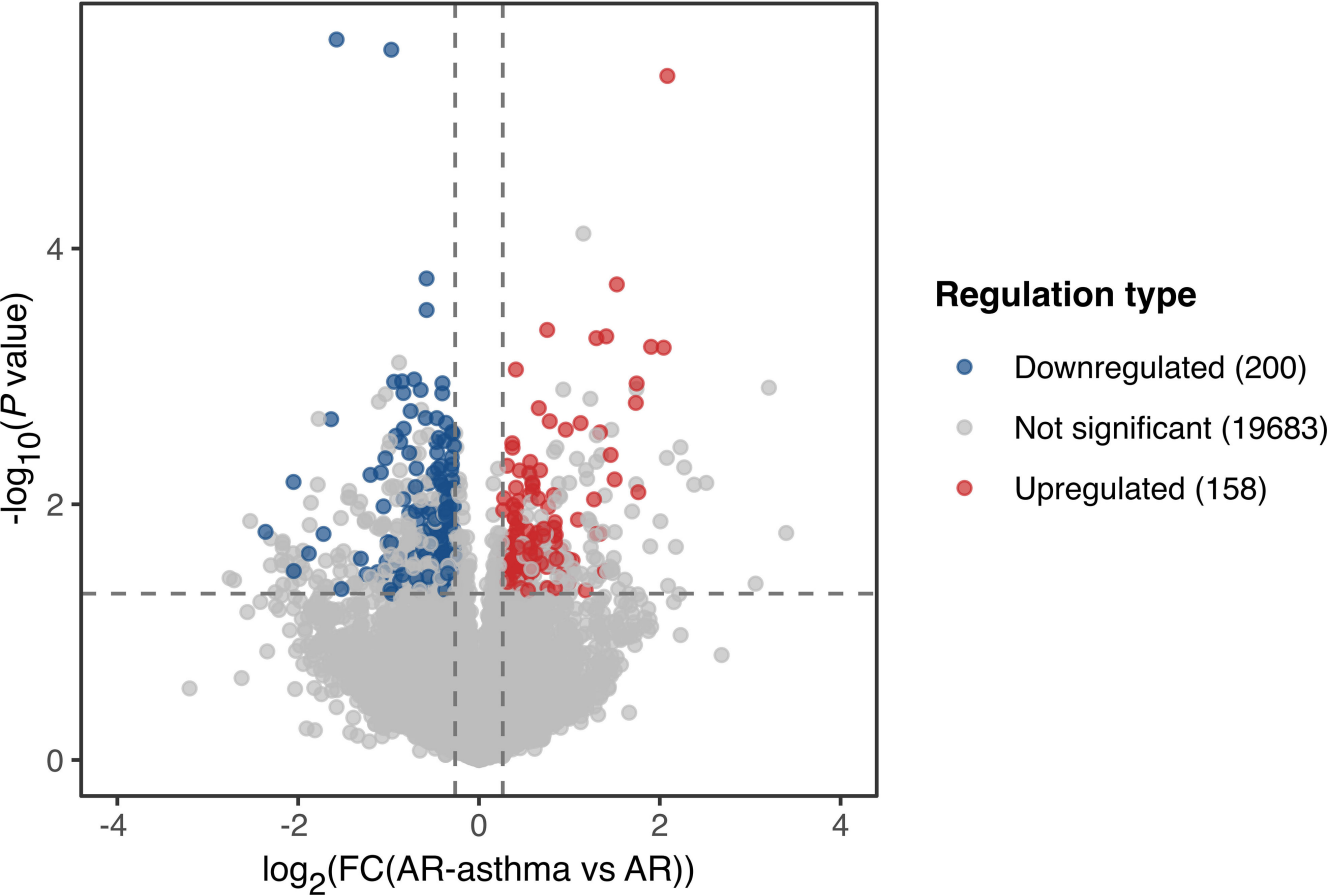
Volcano plot showing DEGs of the AR-asthma group compared to the AR group during the pollen season. Significantly up- and down-regulated genes (|fold change| ≥ 1.2, P value < 0.05 and average FPKM, fragments per kilobase of transcript per million fragments mapped ≥ 1) were labeled as red and blue dots, respectively. AR, allergic rhinitis; DEGs, differentially expressed genes; FC, fold change.

**Figure 2:**
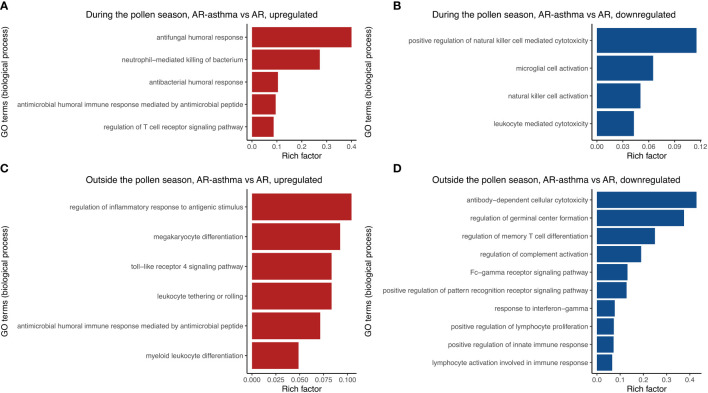
Immune-related GO terms enriched from up-regulated DEGs between the AR-asthma group and AR group during the pollen season **(A)**. Immune-related GO terms enriched from down-regulated DEGs between the AR-asthma group and AR group during the pollen season **(B)**. Immune-related GO terms enriched from up-regulated DEGs between the AR-asthma group and AR group outside the pollen season **(C)**. Immune-related GO terms enriched from down-regulated DEGs between the AR-asthma group and AR group outside the pollen season **(D)**. AR, allergic rhinitis; DEGs, differentially expressed genes; GO, gene ontology.

Transcriptomic RNA sequencing and bioinformatics analysis showed that during the weed pollen season when all the patients had allergic symptoms, 158 genes were upregulated and 200 genes were downregulated in the AR-asthma group compared to the AR group (**Figure 1**). GO enrichment analysis of these DEGs was conducted and the GO terms related to immune reactions were screened out. The following five immune-related GO terms with a *P* value < 0.05 were enriched from the 158 upregulated genes and ordered based on the rich factor: antifungal humoral response, neutrophil-mediated killing of bacterium, antibacterial humoral response, antimicrobial humoral immune response mediated by antimicrobial peptides, and regulation of the T cell receptor signaling pathway. Four immune-related GO terms with a *P* value < 0.05, which included positive regulation of natural killer cell-mediated cytotoxicity, microglial cell activation, natural killer cell activation, and leukocyte-mediated cytotoxicity, were enriched from the 200 downregulated genes and ordered based on the rich factor (**Figure 2**).

### Transcriptomic differences between the AR-asthma and AR groups outside the pollen season

**Figure 3:**
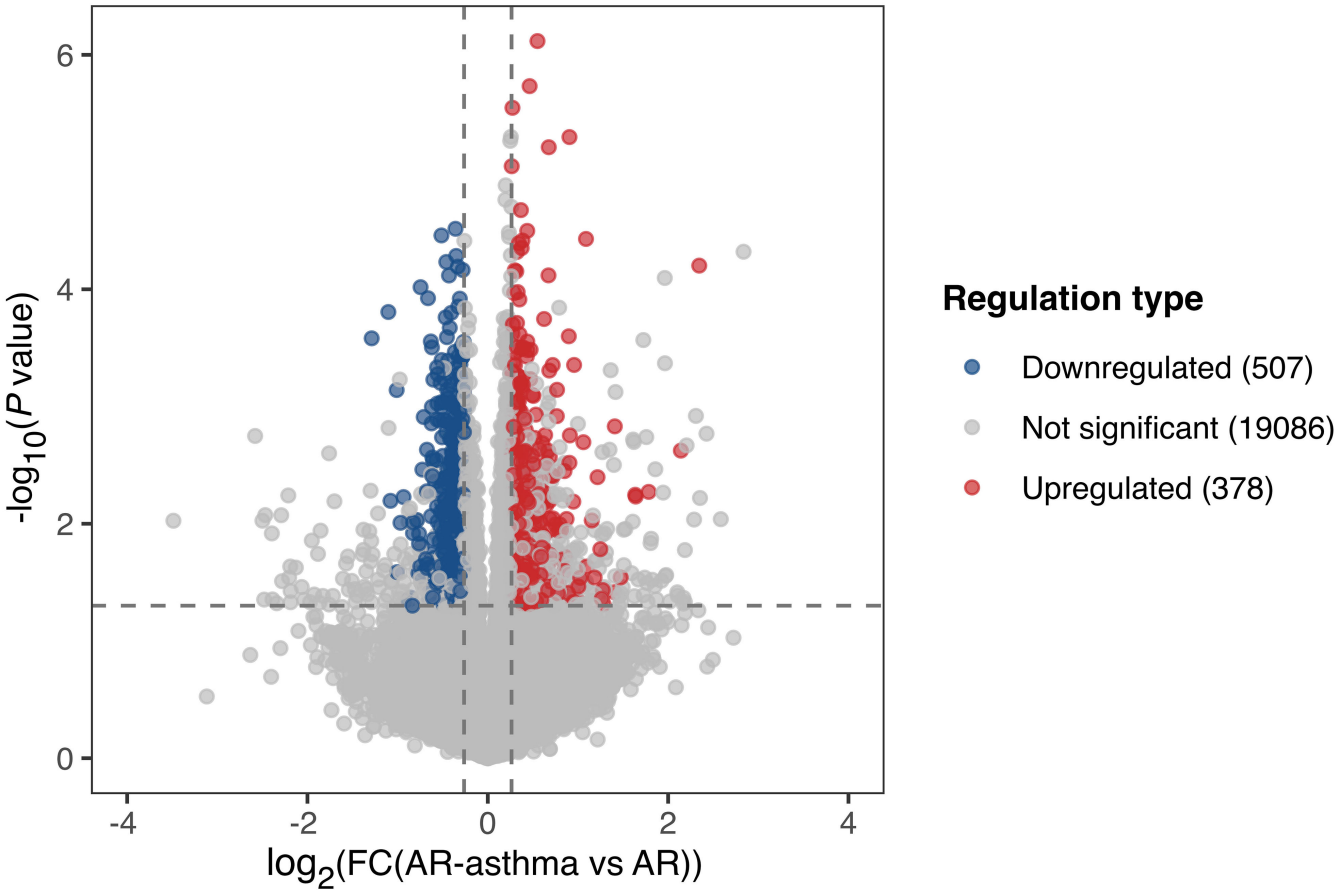
Volcano plot showing DEGs of the AR-asthma group compared to the AR group outside the pollen season. Significantly up- and down-regulated genes (|fold change| ≥ 1.2, P value < 0.05 and average FPKM, fragments per kilobase of transcript per million fragments mapped ≥ 1) were labeled as red and blue dots, respectively. AR, allergic rhinitis; DEGs, differentially expressed genes; FC, fold change.

During winter (outside the weed pollen season), when all the patients had no symptoms, 378 genes were upregulated and 507 genes were downregulated in the AR-asthma group compared to the AR group (**Figure 3**). GO enrichment analysis of these DEGs was also performed. Six immune-related GO terms with a *P* value < 0.05 were enriched from the 378 upregulated genes, and the top five based on the rich factor were as follows: regulation of inflammatory response to antigenic stimulus, megakaryocyte differentiation, toll-like receptor 4 signaling pathway, leukocyte tethering or rolling, and antimicrobial humoral immune response mediated by antimicrobial peptides. Thirty-six immune-related GO terms with a *P* value < 0.05 were enriched from the 507 downregulated genes, and the top five based on the rich factor were antibody-dependent cellular cytotoxicity (ADCC), regulation of germinal center formation, regulation of memory T cell differentiation, regulation of complement activation, and the Fc-γ receptor signaling pathway (**Figure 2**).

### Quantitative PCR validation

**Figure 4:**
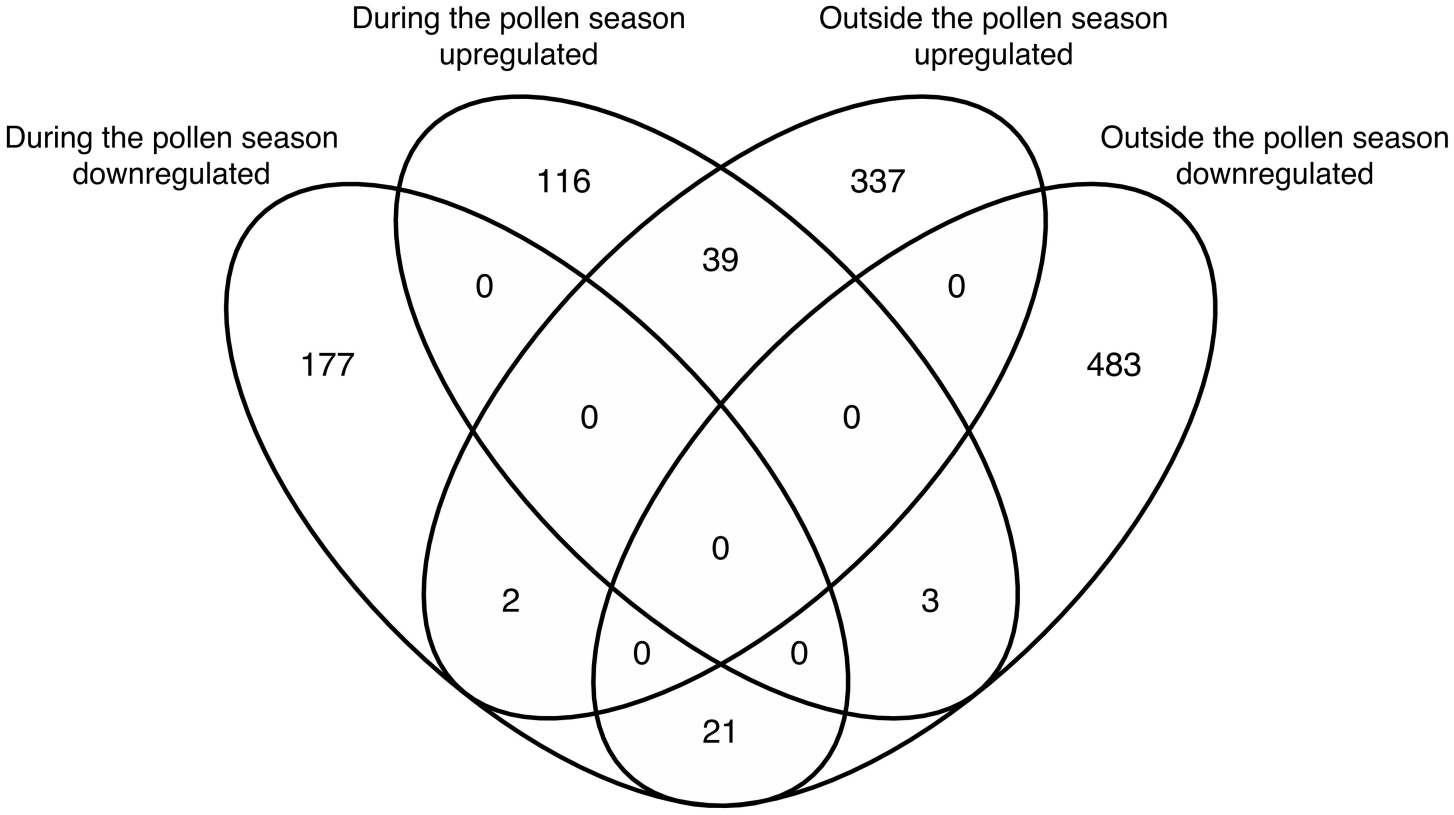
Venn diagram showing the intersection of DEGs between the AR-asthma group and AR group during the pollen season and outside the pollen season. AR, allergic rhinitis; DEGs, differentially expressed genes.

The above-mentioned results indicated that the GO term of antimicrobial humoral immune response mediated by antimicrobial peptides was upregulated in the AR-asthma group compared to the AR group both during and outside the pollen season. The results also showed that if the DEGs between the two groups identified during and outside the pollen season were intersected, 39 DEGs were upregulated and 21 were downregulated at both time points (**Figure 4**). Only one immune-related GO term— the antimicrobial humoral immune response mediated by antimicrobial peptides— could be enriched from the 39 upregulated DEGs. This immune-related GO term included three DEGs, namely: LTF, PF4, and ELANE. However, no immune-related GO term was enriched from the 21 downregulated DEGs.

**Figure 5:**
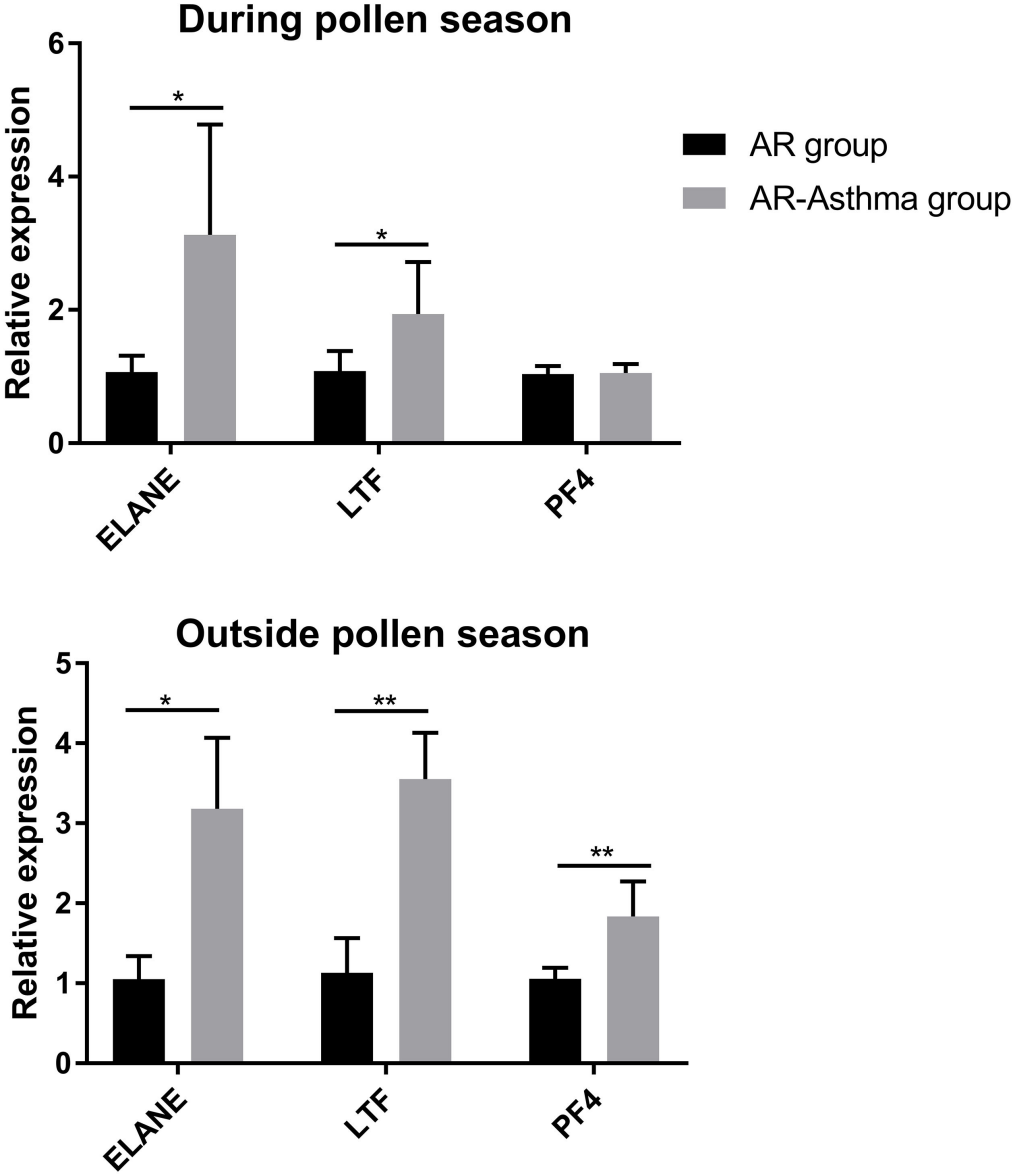
Quantitative PCR validation of three key DEGs between the AR-asthma group and AR group during the pollen season and outside the pollen season. AR, allergic rhinitis; DEGs, differentially expressed genes; PCR, polymerase chain reaction; *P < 0.05; **P < 0.01.

Based on the above results, LTF, PF4, and ELANE were considered possible key DEGs between the AR-asthma and AR groups, and their expression levels were validated through qPCR. The results of the qPCR showed that during the weed pollen season, the expression levels of ELANE and LTF in the AR-asthma group were significantly higher than those in the AR group, whereas the expression levels of PF4 in the two groups were not significantly different. Outside the weed pollen season, the expression levels of LTF, PF4, and ELANE in the AR-asthma group were significantly higher than those in the AR group (**Figure 5**).

## Discussion

In this study, we compared the transcriptome features between the AR-asthma group and the AR group and found that the GO term, antimicrobial humoral immune response mediated by antimicrobial peptides, was upregulated in the AR-asthma group compared to the AR group during and outside the pollen season. Most DEGs of this term, including DEFA3, DEFA4, ELANE, and LTF, encode antimicrobial peptides in neutrophils, indicating that the upregulated antimicrobial immune response in the AR-asthma group is mainly mediated by neutrophils. The results also showed that the GO term of neutrophil-mediated killing of bacterium was upregulated in the AR-asthma group during the pollen season. The important role of airway mucosal neutrophils in the pathogenesis of allergic asthma has been reported in several studies. In animal models of allergic asthma, allergen challenge is associated with the recruitment of neutrophils to the airway mucosa (24, 25). In addition, the interaction between pathogenic allergens and IgE/FcϵRI on the surface of neutrophils in patients with asthma could promote the secretion of neutrophil products, including elastase, myeloperoxidase, and reactive oxygen species (26–28), which induce epithelial cell damage and exacerbate airway mucosal inflammation (29). Impediment of neutrophil recruitment to the airway in asthmatic mice reduces eosinophil infiltration and Th2 cytokine levels, whereas supplementation of neutrophils restores type 2 inflammation and airway hyper-responsiveness (30). However, the difference in neutrophil-mediated immune reactions between patients with AR only and those with AR and asthma has not been widely discussed. It is well known that the development of both AR and allergic asthma are closely associated with increased levels of IgE antibodies and eosinophil inflammation, and that the difference in airway mucosal inflammation in the two conditions may be related to neutrophil-mediated immune response (18). It was reported that patients with allergic asthma showed higher levels of neutrophils and IL-8 in the sputum and more intensive neutrophil chemotaxis than patients with AR without asthma, both at baseline and after bronchial allergen challenge (15, 31). Our findings are consistent with the above-mentioned results, which suggest that the pathway related to neutrophil-mediated antimicrobial immune response is upregulated in patients with AR with asthma compared to patients with AR without asthma, both prior to the weed pollen challenge (outside the pollen season) and during the pollen challenge (during the pollen season).

The enrichment analysis of downregulated DEGs in the AR-asthma group compared to the AR group showed that during the pollen season, the GO terms related to NK cell activation and NK cell-mediated cytotoxicity were downregulated in the AR-asthma group, whereas outside the pollen season, the GO term ADCC was downregulated in the AR-asthma group. ADCC reactions are mainly mediated by NK cells, suggesting that the top downregulated GO terms during and outside the pollen season are consistent and closely related to the activities of NK cells. Compared to the AR group, the AR-asthma group showed depressed cellular cytotoxicity mediated by NK cells. It has been reported that the number and activities of NK cells in patients with asthma are downregulated relative to those in healthy individuals, which is consistent with our findings (32). Duvall et al. (33) reported that the number of NK cells in the bronchoalveolar lavage fluid (BALF) of patients with asthma is lower than that in the BALF of healthy subjects. Also the cytotoxicity of NK cells reflected by killing of K562 myeloid target cells in patients with asthma is impaired compared to that in healthy controls. However, studies on the differences in NK cell activity between patients with asthma and those with AR are lacking. The results of the present study provide new evidence of the role of decreased NK cell-mediated cellular cytotoxicity in the progression from AR to asthma.

It has been reported that ELANE and PF4 are closely related to the pathogenesis of asthma. ELANE is a gene that encodes the protein elastase, which is a serine protease mainly found in neutrophils (34) and can damage the integrity of the airway epithelium (32). Weng et al. (35) found that neutrophil elastase (NE) induces the expression of eosinophil chemokines, thus promoting eosinophil infiltration and type 2 inflammation. In patients with asthma, the level of elastase is significantly correlated with the proportion of neutrophils in the sputum and negatively correlated with the forced expiratory volume in one second (34). PF4 encodes platelet factor 4, which is produced from activated platelets and is increased in the peripheral blood or BALF of patients with asthma (36). Additionally, platelet factor 4 has been found to induce airway hyper-responsiveness in an asthma model (37, 38).However, studies on the comparison of the expression levels of ELANE or PF4 between patients with AR and those with asthma are rare. In the present study, the AR-asthma group showed upregulated ELANE and PF4 expression compared to the AR group in the transcriptomic analysis, suggesting that increased elastase and platelet factor 4 may contribute to the development of allergic asthma from AR. We notice that in the qPCR tests, the measured expression level of PF4 in the AR group and AR-asthma group during the pollen season were not significantly different, and this might be related with the partial degradation to varying degrees in different RNA samples after several months of storage.

The protein encoded by LTF is lacto-transferrin, which is derived from the granules of neutrophils. Lacto-transferrin can inhibit eotaxin-stimulated eosinophil migration into the airway (39) and prevent the development of mucin-producing cells (40). Kruzel et al. found that lacto-transferrin relieved pollen-induced allergic airway inflammation (40). Thus, the phenomenon observed in the present study, which is the higher expression of LTF in the peripheral blood cells of patients in the AR-asthma group than in those of the AR group, may be the protective reaction of the body against asthma inflammation and self-balance. Tsokos et al. (38) reported that patients with asthma show enhanced lacto-transferrin expression in their pulmonary tissues during fatal asthma attacks compared to controls, which is consistent with the findings of the present study.

This study had some limitations. First, the sample size is relatively small, which may lead to bias. In this condition the heterogeneity among different patients may have interfered with the data analysis. Second, there are no healthy controls in this study, and the transcriptomic differences between the AR-asthma group and healthy subjects will provide more supportive evidence for the current findings. Third, we did not validate the results of RNA sequencing and qPCR with specific protein quantitation using western blot assay.

This study focused on the GO terms related to immune reactions while analyzing the difference of transcriptome data between AR and asthma group, and found that the activation of the antimicrobial immune response mediated by neutrophils and the depression of cytotoxicity mediated by NK cells may be involved in the development of asthma from AR. However, prospective studies should be conducted in the future to verify the current findings, in which patients with only AR are recruited and followed up for years until asthma develops. The essential factors involved in the development from AR to asthma could be confirmed through the pair comparison between the AR stage and AR-asthma stage in the same person. Also further experiments in protein level and in animal models are needed to investigate the underlying specific mechanism in the future.

## Data availability statement

The data presented in the study are deposited in the Genome Sequence Archive for Human repository of National Genomics Data Center of China, accession number HRA002991.

## Ethics statement

The studies involving human participants were reviewed and approved by Peking Union Medical College Hospital Review Board. The patients/participants provided their written informed consent to participate in this study.

## Author contributions

We claim that we have directly participated in the planning, execution and analysis of the study. KG designed the project and guided the study. LL, ZW, LC, and YX contributed to the patient recruitment and quantitative PCR. LL, HZ, XW, and KG contributed to the transcriptomic RNA sequencing and bioinformatics analysis. LL wrote the manuscript. ZW, LC, YX, HZ, XW, and KG revised the manuscript. All authors contributed to the article and approved the submitted version.
